# Targeting Intracellular Cholesterol is a Novel Therapeutic Strategy for Cancer Treatment

**DOI:** 10.4172/1948-5956.1000316

**Published:** 2014-12-20

**Authors:** Chandi C Mandal, Md Mizanur Rahman

**Affiliations:** 1 Department of Biochemistry, School of Life Sciences, Central University of Rajasthan, Rajasthan-305817, India; 2 Department of Medicine, University of Texas Health Science Center at San Antonio, San Antonio, Texas-78229, USA

**Keywords:** Intracellular cholesterol, Serum cholesterol, Statins, Cancer growth, Metastasis, microRNA

## Abstract

Substantial data from cell culture and animal studies evidence the preventive effect of statins, cholesterol lowering-drugs, in regulation of cancer cell proliferation and metastasis. Various clinical studies also support this correlation between use of statin and the reduction of cancer incidence. However, in some cases, statins have failed to decrease the risk of cancer. Since, instead of serum cholesterol, intracellular cholesterol may play a crucial role in the regulation of tumorigenesis and metastasis. The mechanism by which cholesterol is stored within cancer cells may differ among cancer types and also in different individuals. This paper discusses the molecular detail to speculate the statin-sensitive cancer. It also highlights that statins may work better as anticancer therapy if it is used with the combination of a specific microRNA (miR).

Cancer continues to be the second highest leading cause of morbidity and mortality throughout the world and is associated with highest economic burden. Overall, this is a matter of grave concern and calls for active research for development of effective and economical treatment strategies to extend the overall survival and improve the quality of life of cancer patients. Since tumor metastasis cause 80% of cancer patient death, metastasis management and control constitute the key requirement to treat cancer patients.

Cancers are perhaps the most complicated diseases because of its genetic heterogeneity and complexity. Each cancer has a distinct type of genetic alteration, oncogenic signaling, metabolic features, and epigenetic changes which are responsible for tumorigenesis [1–5]. Moreover, one sub-population of tumor cells may have a specific type of genetic feature and pathophysiology, which differ highly from other subsets within the same tumor and tumor type. More recently, obesity, diabetes, and hypercholesterolemia are being considered as important risk factors for cancer [6–9]. Emerging data also show the involvement of high-glucose and high-cholesterol in rewiring of metabolic programming which augments the process of tumorigenesis [10–14]. For example, enhanced level of low-density lipoprotein (LDL)- and high-density lipoprotein (HDL)-cholesterol was found in cancer patients [15]. However, the relationship between serum cholesterol and increased risk of cancer still remains obscure [9,16]. Therefore, instead of serum cholesterol, recent studies are focusing toward the key role of intracellular cholesterol in cancer progression and metastasis. For instance, accumulation of intracellular cholesterol was found to be more in tumor tissues [17–19]. Moreover, metastatic cancer cells contain a higher intracellular lipid droplets when compared to normal epithelial cells [20]. Experimental evidences also support the idea that the intracellular cholesterol positively influences proliferation, migration, and invasion of cancer cells [21,22]. This establishes a positive link between elevation of intracellular cholesterol and increased risk of tumorigenesis. But the mechanisms need to be elucidated. Elevated level of cholesterol-rich lipid rafts or microdomains which organize signaling molecule and transduce intracellular signaling within the cells, was found in the plasma membrane of cancer cells [23] and the depletion of cholesterol from these lipid rafts enhances apoptotic death of cancer cells and sensitivity to chemotherapy [24]. Literature also discusses the possibility that the lipid rafts containing high level of cholesterol and GPI-anchored alkaline phosphatase enzyme could be pinched out from the plasma membrane and may form matrix vesicles within cells [25]. These vesicles deposit calcium hydroxy appetite crystal in the extracellular surface, which results in microcalcification of breast cancer tissues [25,26]. Interestingly, microcalcification was also found in other cancers such as ovarian and prostate cancers. New emerging data show a positive association of microcalcification with the malignancy of cancer [27–29]. Thus, elevated level of cholesterol present in the microdomain may promote metastasis of cancers by increasing microcalcification. Recent report shows that 27-hydroxy cholesterol is synthesized from cholesterol within cancer cells, and it may increase breast cancer growth and metastasis, since 27-hydroxy cholesterol binds to estrogen receptor alpha to activate oncogenic estrogen signaling [30]. The expression of cytochrome p450 CYP27A1 enzyme which converts cholesterol to 27-hydroxy cholesterol was shown to be more in epithelial breast tumors, and its expression is positively associated with the tumor grade [31]. These studies highlight the mechanism by which cholesterol may aggravate cancer growth and metastasis in case of breast cancer. However, this mechanism might not be operative to other cancer types or estrogen receptor negative tumor cells. Therefore, further research is required to establish the basic mechanism of cholesterol-mediated cancer growth and metastasis.

Statins are often prescribed to patients for lowering serum cholesterol level. Apart from cardio protective role, statin may prevent osteoporosis by increasing osteoblast differentiation and/or by decreasing osteoclast activity [32,33]. Statins are known to inhibit cholesterol biosynthesis by blocking the activity of 3-hydroxy 3-methylglutaryl COA reductase (HMGCR), the rate limiting enzyme of mevalonate pathway [34]. Many investigators including us have established a preventive role of statins in cancer growth of many cancer types including breast, prostate, and ovarian cancers, evidenced by cell culture and animal model experiments [35–38]. We and others have also recently documented the preventive effect of statin in cancer metastasis, as demonstrated in breast cancer cells induced metastatic mice model [36,39]. Moreover, various clinical studies also support this correlation between use of statin and the reduction of cancer incidence [40–42]. A case-control study of half a million patients had displayed a 48% reduction in renal cell carcinoma [43], and a significant reduction in hepatocellular and in esophageal cancer was observed [44,45] in case of the statin users. However, systematic review of randomized trials had failed to show cancer risk reduction after statin use and showed contradictory findings, with increased incidence for certain cancers and reduced incidence for other types [46,47]. A recent study shows that statin use in Danish cancer patients is associated with reduced cancer-related mortality for 13 cancer types [48], but several investigators have raised their arguments against this report [49,50]. This poses a big challenge to the researchers and it has become imperative to identify the reasons behind the inconsistent results of statin treatment.

The mechanism by which cholesterol is accumulated within tumor cells may vary among cancer types and also in different individuals of same cancer type. In general, acquisition of intracellular cholesterol is mainly carried out in three main ways [17,20]. Excessive accumulation of cholesterol within cells could be either due to increased LDL-cholesterol internalization because of high expression of low density lipoprotein receptor (LDLR), and/or due to increased synthesis of cholesterol inside cancer cells because of high activity of HMGCR, and/or inhibition of cholesterol efflux due to deficiency or inactivation of ATP-binding cassette (ABC) transporter proteins such as ABCA1 and ABCG1 [17,20]. For instance, abnormal expressions of LDLR and HMGCR have been found in many cancer types [17,18]. Moreover, reciprocal expressions of LDLR and HMGCR in cancer tissues have also been documented [22]. Thus, expression patterns of LDLR and HMGCR may vary between statin-sensitive cancers and statin-insensitive cancers. Detailed molecular investigations of individual tumors could explain the reason for enrichment of intracellular cholesterol inside cancer cells.

## Therapeutic Aspects: Targeting Intracellular Cholesterol

Accumulating evidences highlight that instead of statin alone, the combination of statin with other partner might serve a better function to prevent cancer growth and metastasis. Several investigators along with ours have shown that omega-3 fatty acids (docosahexaenoic acid; DHA and ecosapentaenoic acid; EPA), active components of fish oil, prevent cancer growth and metastasis, evidenced by cell culture and animal experiments [19,51–53]. Clinical studies also document the preventive role of omega-3 fatty acids in cancer risk [14]. Omega-3 fatty acids lower serum triacyl glycerol (TAG) which promotes tumorigenesis. Beside these, omega-3 fatty acids also prevent cholesterol synthesis in tumor cells [54]. Moreover, omega-3 fatty acids especially DHA disorganize the lipid rafts of plasma membrane by displacing cholesterol molecules, and dampen the microdomain mediated signaling, which may prevent cholesterol-assisted tumorigenesis [55,56]. This seems that the combined therapy of omega-3 fatty acid might increase the effectiveness of statin in preventing cholesterol-induced cancer progression and/or metastasis.

In the last decade, many researchers have worked to establish the role of microRNA (miRNA) in cancer progression and metastasis [57]. Dysregulation of miRNAs not only affects various physiological functions but also promotes many pathological functions. In fact, some miRNAs [e.g., miR-122 (Clinicaltrials.gov number, NCT01200420), miR-34 (Clinicaltrials.gov number, NCT01829971)] are currently underway in clinical trials [58,59]. Thus, miRNA seems to be a promising therapy in near future. Recent evidences indicate the involvement of many miRNAs (e.g., miR-33a, miR-128, miR-145, miR-185, and miR-19b) in the regulation of cholesterol metabolism [60–63]. Moreover, forced expression of miR-33a in cell lines directly suppresses the expression of ABCA1, resulting in inhibition of cholesterol efflux, whereas antagomir of miR-33a (anti-miR, inhibitor) increases cholesterol efflux by increasing the expression of ABCA1. But the combination of statin with anti-miR-33a may not be good choice for cancer treatment, because statin treatment upregulates miR-33a expression, and moreover, miR-33 inhibits cell proliferation and cell cycle progression by targeting CDK6 and cyclin D1 [60,64]. Similarly, overexpression of miR-128 upregulates sterol-regulatory element-binding protein 2 (SREBP2) which transcriptionally increases expressions of HMGCR and LDLR, and inhibits expressions of transporter ABCA1 and ABCG1 [61]. These studies have shown an enhancement of LDLR by overexpressing of this miRNA whereas inconsistent results were found in case of HMGCR. It was demonstrated that miR-128 expression increases cellular cholesterol. Nevertheless, anti-miR-128 could not be used as an anticancer therapy, since overexpression of miR-128 have been shown to inhibit functional activity of tumor suppressor protein p53 [65]. However, forced expression of miR-185 and miR-372 inhibits SREBP1 and SREBP2, and its downstream target HMGCR with concomitant decrease of proliferation, migration, and invasion of prostate cancer cells [62]. Recent findings also show that miR-19b inhibits transporter ABCA1 with a concomitant decrease of cholesterol efflux, and it also promotes tumor growth and metastasis by blocking p53 activity [63,66]. Altogether, these data herein, recapitulate that mimic of miR-185, miR-372 and antagomir of miR-19b could be good candidates for cancer therapy. Thus, such combination of statin with either miR-185, or miR-372, or anti-miR-19b may be proposed for better therapy to prevent cancer growth and metastasis.

In brief, this note just provides a concept, but extensive research is required to understand the molecular mechanism as to how cholesterol regulates cancer progression and metastasis, and to determine if these microRNAs and such combinations work better for anticancer therapy.
